# Trans-hepatic Lung Ultrasound – A Window for Supine Patients

**DOI:** 10.24908/pocus.v6i1.14753

**Published:** 2021-04-22

**Authors:** Miguel Lourenço Varela, Sofia Branco Ribeiro, Andriy Krystopchuk, Daniel Nunez

**Affiliations:** 1 Intensive Care Medicine 1, Hospital de Faro, Centro Hospitalar Universitário do Algarve Faro Portugal

**Keywords:** POCUS, lung ultrasound, trans-hepatic

## POCUS Protocol

Lung ultrasound has gained increasing use in the last few years, especially in the critically ill patients. By applying the probe on the thorax, much of the lung can be inspected and multiple conditions can be diagnosed and monitored, through anterior, lateral and posterior thoracic views 1.

However, the majority of ICU patients are supine, barring access to visualization of the lowest and most posterior portion of the lower lobe, as it extends through most of the posterior half of the lung and only little on the lateral surface 1, 2. Visualization of the lower lobe is important, as it occupies a significant portion of the right lung and it is usually affected in aspiration pneumonia 3.

**Figure 1:**
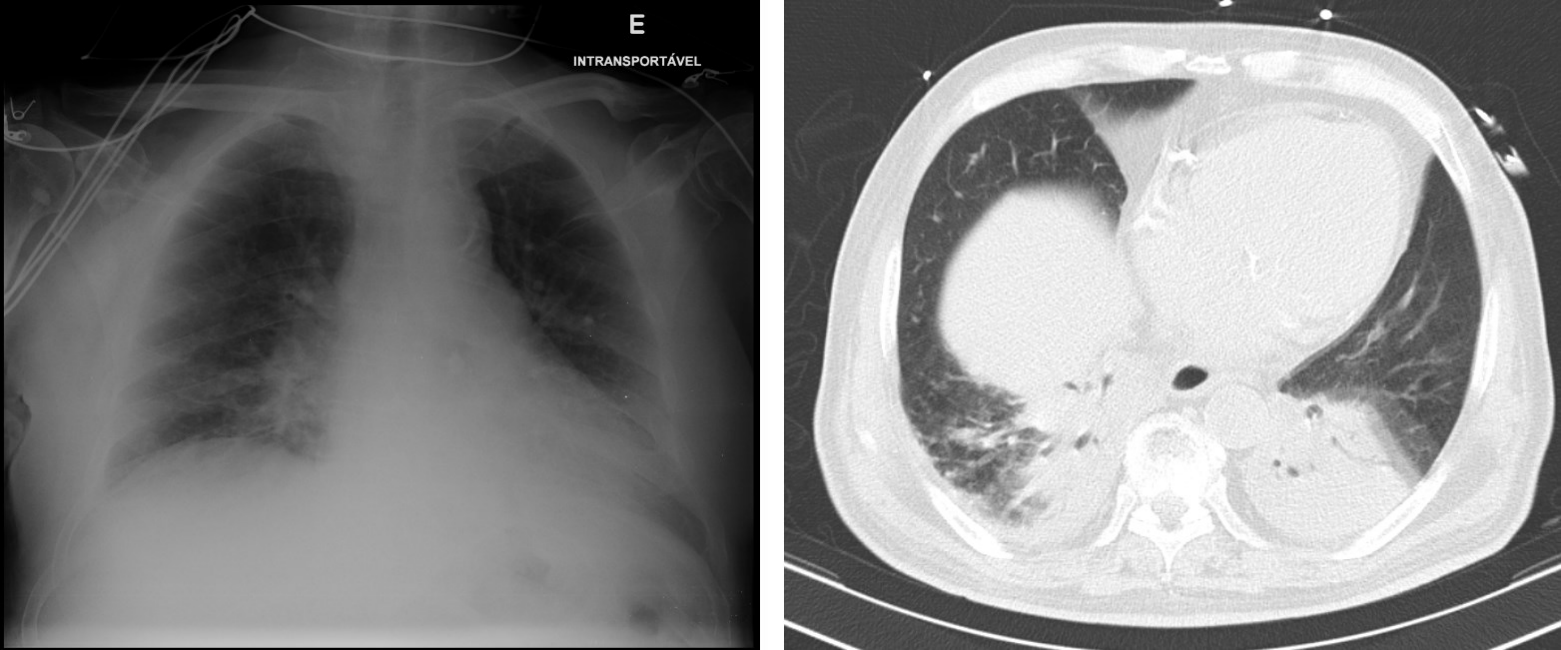
Chest x-ray of the patient (left) and CT scan of same patient (right), performed in the same day, showing right lower lobe consolidation, as well as, left lower lobe consolidation.

We describe a novel approach to visualization of part of the lower lobe of the right lung, through a trans-hepatic approach. We first used this approach in a 72-year-old patient with dyspnea and severe hypoxemia, to diagnose a lower lobe pneumonia, which was ill-defined in the chest x-ray and later confirmed by looking at a computed tomography (CT) (Figure 1). This later exam had been ordered due to the suspicion of pulmonary embolism, since the initial chest x-ray had not revealed a condensation. Use of this novel trans-hepatic window could have made this diagnosis possible earlier. The lower lobe pneumonia was also not seen in the standard anterior and lateral views and a complete posterior view was not possible as the patient was supine and had difficulty in lateralizing.

**Figure 2:**
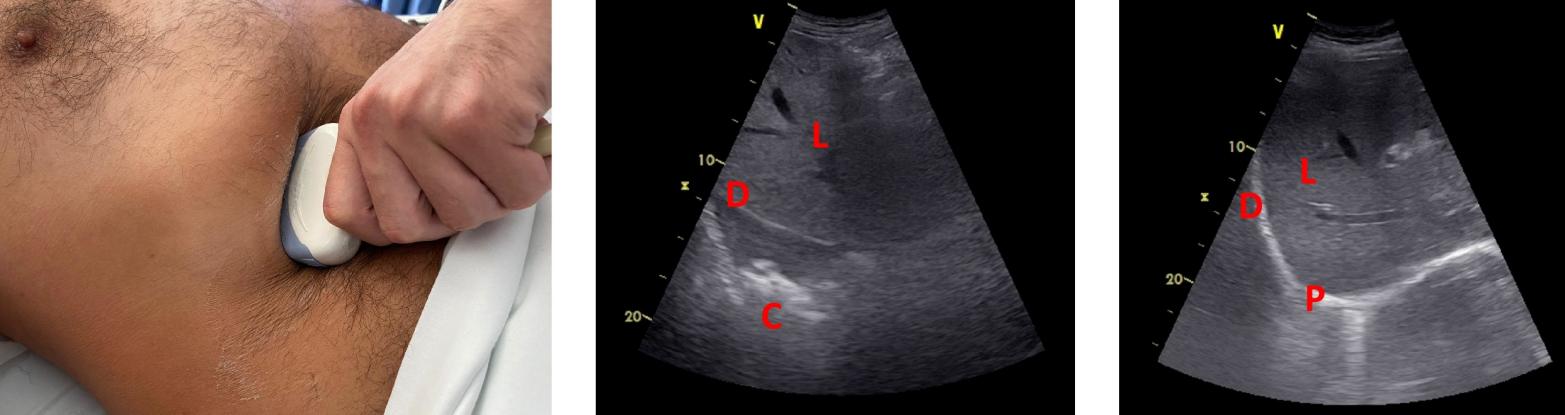
Positioning of the ultrasound probe in order to perform a trans-hepatic lung ultrasound (left panel); lung ultrasound findings in the same patient, showing the liver (L), diaphragm (D) and aconsolidated lower lobe of the right lung (C), as shown by the shred sign (middle panel); lung ultrasound findings in another patient without pneumonia, with visible pleura (P) and lung sliding (right panel).

**Figure 3:**
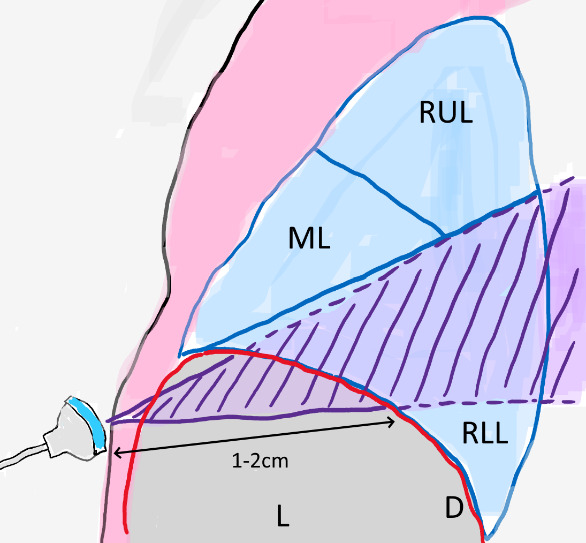
Schematics of probe position relative to the liver (L), average depth to visualize the diaphragm (D) and the right lower lobe (RLL); part of the RLL may not be seen depending on patient anatomy; the middle lobe and the right upper lobe are too deep to be visualized, except if atelectasis is present.

This window can be obtained by placing a curvilinear probe transversely below the liver border and tilting the probe up until the liver and diaphragm can be seen (Figure 2, left panel); the diaphragm dome will be seen as an hyperechogenic line above the liver and, superior to it, the lowest portion of the inferior lobe will be presented (Figure 2, middle panel, and Video 1). The lower lobe will be visible in case a pneumonia or atelectasis is present. Identifying the shred sign will allow for the diagnosis of right lower lobe pneumonia. In patients without pneumonia, the pleura and lung sliding will be visible (Figure 2, right panel, and Video 2). In our brief experience, this window may not be attainable in obese patients, those with hepatomegaly or with ascitis, as the lowest portion of the lung becomes too deep relative to the liver border for the ultrasound to penetrate. Thus, this technique is not sensitive enough for excluding the diagnosis of right lower lobe pneumonia. A schematic showing the relative position to the ultrasound probe of the various organs is shown in Figure 3.

We believe this approach should be included in the standard approach to lung ultrasound, as it is quickly and easily obtained, without having to lateralize the potentially unstable patient.

## Statement of Ethics

The requirement for informed consent was waived, as the information presented does not allow for patient identification.

## Conflicts of interest

The authors declare no conflicts of interest. 

## Supplementary Material

Video S1 Lung ultrasound findings, showing the liver, diaphragm, and a consolidated lower lobe of the right lung, as shown by the shred sign.

Video S2 Lung ultrasound findings in a patient without pneumonia, with visible pleura and lung sliding.
